# The Delivery of RNA-Interference Therapies Based on Engineered Hydrogels for Bone Tissue Regeneration

**DOI:** 10.3389/fbioe.2020.00445

**Published:** 2020-05-12

**Authors:** Tingting Yu, Hufei Wang, Yunfan Zhang, Xing Wang, Bing Han

**Affiliations:** ^1^National Engineering Laboratory for Digital and Material Technology of Stomatology, Beijing Key Laboratory of Digital Stomatology, Department of Orthodontics, Peking University School and Hospital of Stomatology, Beijing, China; ^2^Beijing National Laboratory for Molecular Sciences, State Key Laboratory of Polymer Physics and Chemistry, Institute of Chemistry, Chinese Academy of Sciences, Beijing, China; ^3^University of Chinese Academy of Sciences, Beijing, China

**Keywords:** RNA interference, bone regeneration, hydrogel, drug delivery, tissue engineering

## Abstract

RNA interference (RNAi) is an efficient post-transcriptional gene modulation strategy mediated by small interfering RNAs (siRNAs) and microRNAs (miRNAs). Since its discovery, RNAi has been utilized extensively to diagnose and treat diseases at both the cellular and molecular levels. However, the application of RNAi therapies in bone regeneration has not progressed to clinical trials. One of the major challenges for RNAi therapies is the lack of efficient and safe delivery vehicles that can actualize sustained release of RNA molecules at the target bone defect site and in surrounding cells. One promising approach to achieve these requirements is encapsulating RNAi molecules into hydrogels for delivery, which enables the nucleic acids to be delivered as RNA conjugates or within nanoparticles. Herein, we reviewed recent investigations into RNAi therapies for bone regeneration where RNA delivery was performed by hydrogels.

## Introduction

RNA interference (RNAi), first observed in the late 1980s by Fire et al. (1998) is an efficient gene silencing therapeutic strategy. This technique enables the post-transcriptional downregulation of disease-related gene expression by using small interfering RNA (siRNA) and microRNA (miRNA) molecules (Gori et al., 2015; Chen X. et al., 2018). Since the discovery of RNAi won the Nobel prize in 2006, billions of dollars have been investigated in this field and a wide range of applications have been used for various therapeutic purposes, including bone regeneration (Liang et al., 2015; Nguyen et al., 2018; Yang et al., 2018). On despite its vast therapeutic potential, RNAi-based clinical trials have encountered obstacles, including immune-related toxicities and insufficient therapeutic efficacy (Kleinman et al., 2008; Davis et al., 2010; DeVincenzo et al., 2010; Bobbin and Rossi, 2016). One of the major issues that impedes RNAi’s translational progress toward clinical usage is how to deliver RNA molecules locally and accurately to enhance efficiency and avoid side effects of RNAi therapy (Krebs and Alsberg, 2011). Three-dimensional biomaterials, such as hydrogels, are prospective tools for the local and controlled delivery of a variety of molecules for disease treatment and tissue engineering applications (Zhang et al., 2019). Some hydrogels have been engineered specifically for RNA delivery to facilitate their therapeutic efficacy (Wang and Burdick, 2017; Wang et al., 2017; Feng G. et al., 2018).

Recently, several reviews have introduced the current progress in RNAi therapy for treating bone related diseases (Ji et al., 2016; Arriaga et al., 2019; Leng et al., 2020), and advancement of designed scaffolds for RNAi delivery *in vitro* or *in vivo* for treating various diseases (Ku et al., 2014; Ho W. et al., 2016; Wang and Burdick, 2017; Singh et al., 2019). However, no study has yet systematically summarized the application of hydrogel-based scaffold as RNAi delivery method for bone regeneration. Herein, this review will discuss the hydrogel-based delivery system, and their advanced design strategies for carrying two types of RNAi molecules, including siRNAs and miRNAs, in the field of bone regenerative medicine.

## Clinical Need for New Bone Regeneration Strategies

Bone defects can be caused by fracture, infection, trauma, tumor resection, or skeletal abnormalities. Nearly 2.2 million bone grafts are performed worldwide annually, and over 20% of patients suffer from delayed healing (Giannoudis et al., 2005; Fillingham and Jacobs, 2016). To date, autologous bone grafts are still the main therapeutic strategy for repairing segmental defects of a critical size (Schemitsch, 2017). The bone is usually harvested from the iliac crest, which is a site that is not weight bearing. However, the weak points of autologous surgery are obvious, including the multiphase operation, post-operative infection after the harvesting procedures, and the possibility of low effectiveness of the grafts (Betz, 2002). Synthetic scaffolds or demineralized bone matrix are substitutes that provide a hospitable environment for new bone formation, but their efficiency and osteogenesis potential are in need of improvement (Betz, 2002).

During skeletal development, signaling molecules, such as bone morphogenetic proteins (BMPs), play important roles in inducing osteoblast differentiation and bone growth (Salazar et al., 2016; Majidinia et al., 2018). BMPs have also been widely used as growth factors for the induction of mesenchymal stem cell (MSC) osteogenesis in bone tissue engineering applications (Ho S.S. et al., 2016; Chen Z. et al., 2018). Because of their extensive bone-induction properties, BMP-based therapy has been approved by the Food and Drug Administration (FDA) in selected indications, such as sinus augmentations and spinal fusions (McKay et al., 2007). In these treatments, recombinant human BMP-2 (rhBMP2) was added to an absorbable collagen sponge (ACS) carrier to induce bone formation. It was reported that the clinical outcomes were equivalent to those of autogenous bone grafts at a 1.5-mg/cc concentration of rhBMP2/ACS. However, as the concentration of endogenous proteins in natural bone was at the ∼ng/ml level, the high dose protein therapy has been found to be associated with a greater apparent risk of new malignancy, wound-related complications, and osteolysis (Cahill et al., 2009; Carragee et al., 2011). Moreover, the high cost of protein products leads to the significant elevation of hospital charges, which might also impede widespread application (Cahill et al., 2009).

The bone microstructure is composed of mineralized extracellular matrix and bone remodeling units. The balance of osteoclasts and osteoblasts consistently helps to maintain bone hemostasis (Datta et al., 2008). Osteocytes, which are located within the bone matrix, are the most abundant cells in bone. MSCs can be differentiated into osteocytes under certain stimuli, and they can obtain the ability to self-renew without losing their multipotency. Based on their superior biological behaviors, MSCs are used as a promising cell source for bone tissue engineering and regenerative medicine (Klimczak and Kozlowska, 2016). MSCs are usually transplanted on scaffolds, and the cells are able to produce an extracellular matrix to induce local bone formation (Heo et al., 2019; Zhang et al., 2019; Zhao et al., 2019). However, cell-based therapy still cannot efficiently regenerate critical-sized bone defects.

## RNAi Mediated Gene Silencing and Its Applications in Bone Regeneration

**FIGURE 1:**
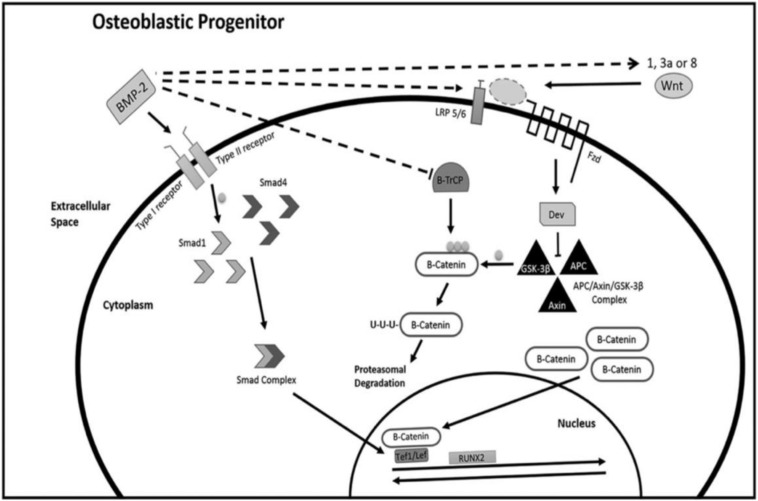
Signaling pathways in osteogenesis. Reprinted from a previous study Arriaga et al. (2019) with permission.

Bone is continuously turning over and remodeling through the actions of bone-resorbing osteoclasts and bone-forming osteoblasts, which originate from hematopoietic and mesenchymal lineages, respectively. The activity of these two types of cells is regulated by several key signaling pathways (Majidinia et al., 2018), including the RANKL pathway, BMP signaling pathway (Katagiri and Watabe, 2016), Wnt signaling pathway (Karner and Long, 2017), and Notch signaling pathway (Rebay et al., 1991). The crosstalk between these signaling pathways helps to maintain the balance between bone resorption and bone formation (Figure 1). RNA molecules, including miRNAs and siRNAs, have been recently discovered as a crucial mechanism in modulating bone remodeling (Lian et al., 2012).

**FIGURE 2:**
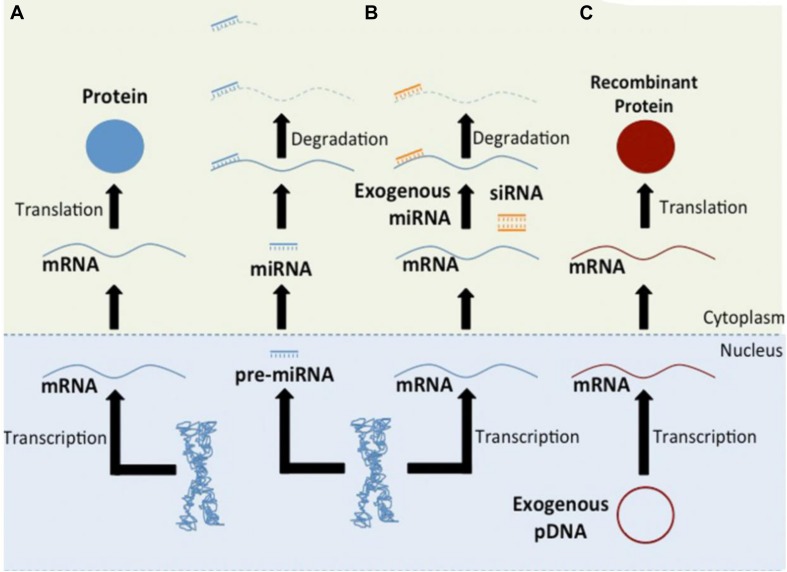
Schematic of gene-based therapies. Pathway **(A)** shows the central dogma of molecular biology. The regulation of protein translation through RNAi is shown in Pathway **(B)**. Endogenous miRNAs, exogenous miRNAs, or synthetic siRNAs can bind mRNA and induce its degradation. Pathway **(C)** shows the production of recombinant proteins. Reprinted from a previous study Rose and Uludag (2013) with permission.

As a single gene can modulate bone formation or bone resorption extensively, gene therapy is particularly applicable to bone tissue regeneration, and upregulating Runt-related transcription factor 2 (Runx2) or BMP expression levels may induce extensive new bone regeneration (Salazar et al., 2016; Liu et al., 2019). To promote osteogenesis, genetic elements can also negatively regulate the expression of proteins that inhibit osteogenesis. The strategy of RNAi-based therapy is to alleviate the disease phenotype by reducing specific disease-related gene expression levels (Lam et al., 2015; Murashov, 2017). Both miRNA and siRNA can bind messenger RNA (mRNA) and induce the degradation of mRNA, and this process can be used to downregulate inhibitors of osteogenesis (Figure 2).

miRNAs are the most studied non-coding RNAs related to bone diseases and bone metabolism. miRNAs are single-stranded RNA molecules composed of 20–24 nucleotides, and they function by silencing the expression of mRNA through binding to complementary sequences in the 3′ untranslated region (UTR) of target mRNAs. Approximately 1,800 miRNAs are encoded by the human genome, and each miRNA is predicted to regulate several target genes (Lewis et al., 2005). It has been concluded through computational predictions that more than 50% of genes are potentially regulated by miRNAs, and this type of regulation exerted by miRNAs has been proven to be reversible (Lewis et al., 2005; Friedman et al., 2009). To date, miRNAs have been demonstrated to be critical regulators participating in various biological processes, including cell differentiation (Chen et al., 2017; Yang et al., 2018), cell death (Liu et al., 2018), cell proliferation (Chen et al., 2017), and metabolism (Vienberg et al., 2017). The variation of miRNAs can affect bone remodeling and can result in bone fractures and osteoporosis (Feng Q. et al., 2018).

To date, a large number of miRNAs have been identified that are involved in the regulation of bone metabolism and serve as specific therapeutic targets for diagnosis and bone disease treatment (Chen et al., 2017; Taipaleenmaki, 2018; Yang et al., 2018). Runx2 is a key regulator of bone development, and it is targeted by a range of miRNAs. In MSCs, miR297a-5p, miR297b-5p, and miR297c-5p were found to reduce MSC osteogenic differentiation potential by targeting the 3′UTR of Runx2 and inhibiting the expression levels of alkaline phosphatase (ALP) and osteocalcin (OCN) (Yang et al., 2018). In osteoblasts, miR542-3p targets bone morphogenetic protein-7 (BMP-7), which results in the suppression of cell differentiation and proliferation (Kureel et al., 2014). However, miR433-3p promoted osteoblast differentiation by targeting dickkopf-1 (DKK-1) (Tang et al., 2017), which is an antagonist of the WNT signaling pathway; the inhibition of DKK-1 upregulated WNT signaling and promoted osteogenic differentiation of cells. Furthermore, miRNAs can also serve as therapeutic targets for bone diseases. It was reported that miR151-5p can effectively ameliorate osteopenia in systemic sclerosis (SSc) by targeting IL-4. Overexpression of miR151-5p in bone marrow mesenchymal stem cells (BMMSCs) was capable of decreasing the IL-4 expression level and inhibiting its induced osteogenic deficiency (Chen et al., 2017). These investigations indicated that miRNAs played an important role in bone remodeling by targeting major genes and signaling pathways related to osteogenic differentiation.

Short interfering RNAs (siRNAs) are used in a subset of RNAi-based approaches and have been increasingly investigated for therapeutic purposes. Similar to miRNA mimics, artificially synthesized siRNAs can lead to gene silencing. siRNAs are double-stranded RNA molecules that can exert gene silencing against a complementary mRNA target after the transfection of the siRNA, which is performed by a method similar to that used for miRNA mimics. However, while miRNAs may target a number of genes at the same time through partial complementarity, siRNAs can only target one specific gene with full complementarity (Lam et al., 2015).

## The Challenges of RNAi Therapy

RNAi methods represent very powerful tools for elucidating gene function, but there are inevitable challenges that need to be overcome to achieve clinical translation. In 2010, unmodified siRNAs were used for the first time in clinical trials, resulting in questionable RNAi effects and immune-related toxicities (Kleinman et al., 2008; DeVincenzo et al., 2010). Later, investigators used systemically administered siRNA nanoparticle systems to achieve great advances, but their therapeutic efficacy was still insufficient (Davis et al., 2010). Recently, the first RNAi drug, patisiran (Onpattro; Alnylam Pharmaceuticals), was introduced for the treatment of hereditary transthyretin amyloidosis (hATTR) and was approved by the US Food and Drug Administration (FDA) on 10 August 2018, which indicated that a new era in RNAi therapy had begun (Adams et al., 2018; Heras-Palou, 2019). Currently, after absorbing the lessons from prior failures and achievements, investigations that applied RNAi to soft tissue diseases, such as kidney, liver, and dermis, have been established in the form of several clinical trials (Ozcan et al., 2015; Zuckerman and Davis, 2015; Bobbin and Rossi, 2016). However, studies on bone-related diseases based on RNAi techniques remain limited.

Transportation of RNAi molecules into specific organs or cells is the main obstacle to RNAi therapeutic development. Naked RNAi molecules are vulnerable when directly injected into tissues or blood and will further cause off-site bioactivities. When transported through cell membranes, RNAi molecules can be repelled because of their like-charged physiochemical property, as miRNA and siRNA have a negatively charged phosphate backbone (Whitehead et al., 2009; Lam et al., 2015). During internalization, siRNAs and miRNAs are exposed to endolysosomal fusion, rapid acidification and degradation, which dramatically affect RNAi delivery efficiency (Kim et al., 2019). Moreover, the large size of RNAi molecules and their hydrophilic properties may also impede the transportation process. Therefore, a promising delivery system is essential for the clinical translation of RNAi therapies.

A successful delivering system should protect RNAi molecules from cellular barriers, target RNAi to a specific type of cells or tissues, and achieve sustained release of RNAi molecules into the cytoplasm. To date, although the delivering method undergoes a rapid development, scaffolds obtain both advantages and limitations in the use of RNAi delivering for bone regeneration, as we discussed in Table 1.

**TABLE 1:** Advantages and disadvantages of using scaffolds as RNAi delivery method for bone tissue engineering.

Advantages	Disadvantages
Delivery platform for RNAs and structural support for infiltrating cells during bone regeneration	The interactions between scaffolds and vectors may limit the release of RNAs
Locally deliver RNAs to specific sites to reduce unwanted off-target effect	Long-term controlled release of RNAs from scaffolds is difficult to be achieved *in vivo*
Release RNAs in a controlled manner	
Protect RNAs from physiological degradation	

## Applications of Hydrogel RNAi Delivery Systems for Bone Tissue Regeneration

A range of biomaterials have been investigated as molecular RNAi carriers for bone tissue engineering, including nanoparticles (Schade et al., 2014; Levingstone et al., 2019), bioactive glass (Kim et al., 2016a, b), multilayer films (James et al., 2019), solid porous scaffolds (Liu et al., 2015; Chen et al., 2017), and hydrogels (Huynh et al., 2018; Nguyen et al., 2018). Hydrogels are 3D hydrophilic networks composed of natural or synthetic polymers and can be designed with diverse mechanical, physical and chemical properties for various applications. In this case, hydrogel-based scaffolds are popular choices as RNAi carriers. They are biocompatible, biodegradable, capable of encapsulating RNAi molecules into specific bone defect sites, and achieving a sustained release to surrounding cells (Wang and Burdick, 2017). The applications of RNAi hydrogel-based scaffolds are summarized in Table 2, and their applications are reviewed in more detail.

**TABLE 2:** The applications of RNAi delivered by hydrogel scaffolds for bone tissue engineering.

Scaffolds	RNA interference	Cell type	Animal models/time points	References
PEG hydrogel	siRNA-Noggin miR-20a	Human mesenchymal stem cell	Calvarial bone defect in rats, 12 weeks.	Nguyen et al., 2018
SFCS scaffolds	siRNA-GNAS1 siRNA-PHD2	Human embryonic stem cell	Subcutaneous transplantation, 10 weeks.	Zoldan et al., 2011
PLGA-PEG-PLA-DM hydrogel scaffolds	siRNA-Cy5	–	Femur fracture model, 4 weeks.	Wang Y. et al., 2018
PLLA scaffolds	siRNA-Sema4d	–	Femur osteoporotic defect model in ovariectomized rats, 8 weeks.	Zhang Y. et al., 2016
PLA-DX-PEG polymer	siRNA-Noggin	–	The dorsal muscle pouches of mouse for ectopic bone formation, 7 days.	Manaka et al., 2011
CS/TPP/hyaluronic acid NPs	Anti-miR-138	Rat mesenchymal stem cells	Calvarial bone defect in rats, 8 weeks.	Wu et al., 2018
PLLA scaffold; HP vector-PLGA microsphere	miR-26a	–	Calvarial bone defect in mouse, 8 weeks.	Zhang X. et al., 2016

*PEG, polyethylene glycol; PEI, polyethyleneimine; SFCS, silk fibroin-chitosan; GNAS1, guanine nucleotide-binding protein alpha subunit 1; PHD2, prolyl hydroxylase domaincontaining protein 2; PEG-PLA-DM, poly (ethylene glycol)-poly (lactic acid)-dimethacrylate; PLLA, poly-l-lactide; Sema4d, semaphorin4d; PLA-DX-PEG, poly-D,L-lactic acid-p-dioxanonepolyethylene glycol block co-polymer; CS, chitosan, TPP, tripolyphosphate, NPs, nanoparticle; HP, Hyperbranched polymer.*

**FIGURE 3:**
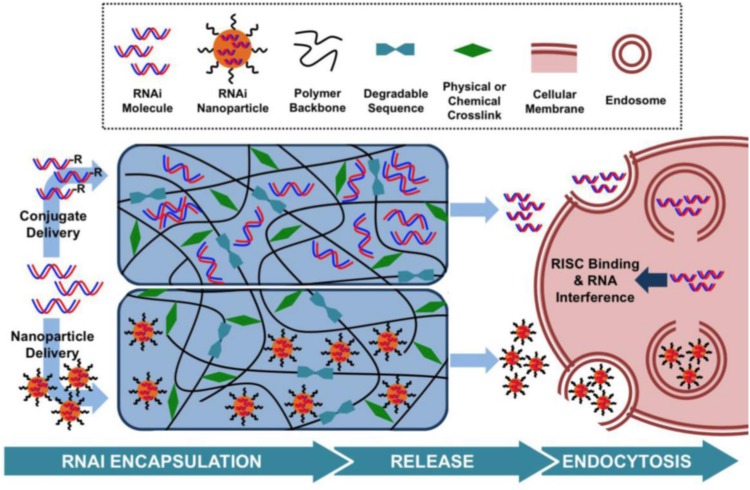
Hydrogel-based RNAi delivery strategies include encapsulation within a nanoparticle or the use of an RNAi conjugate. Degradable sequences, varied polymer charges, and crosslinking mechanisms control the rate of RNAi molecule release. Upon release, nanoparticles or RNAi molecules are able to interact with cell membranes and enter the cell, leading to gene silencing. Reprinted from a previous study Wang and Burdick (2017) with permission.

To improve RNAi uptake from hydrogels and protect the RNA molecules from enzymatic hydrolysis, numerous strategies have been investigated, including inclusion within nanoliposomes to produce lipophilic nanoparticles or ionic complexation with cationic polymers, such as polyethyleneimine (PEI) (Tamura and Nagasaki, 2010; Kim et al., 2014; Zhang X. et al., 2016). To date, hydrogels have been used as carriers in which nucleic acids have been delivered as RNA conjugates or within nanoparticles (Wang and Burdick, 2017; Figure 3).

**FIGURE 4:**
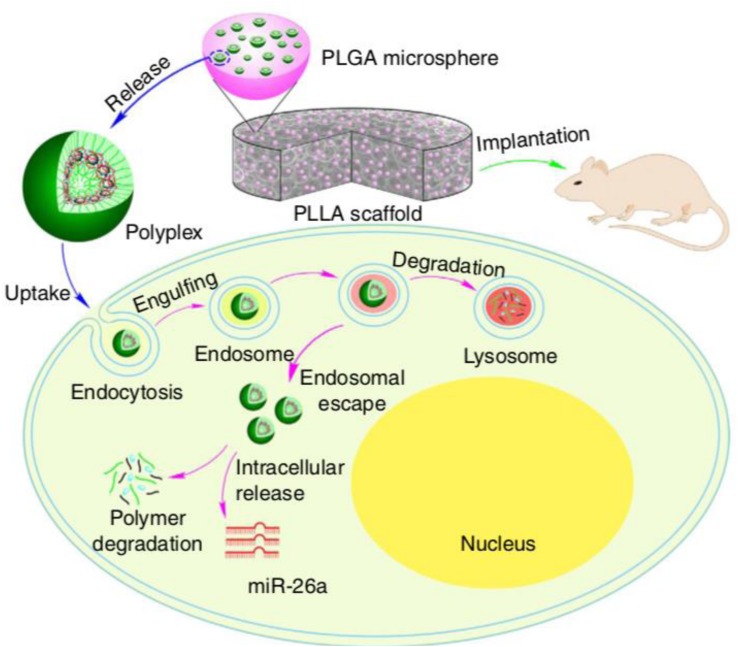
Two-stage delivery of miRNA from PLGA microspheres immobilized on a nanofibrous (NF) scaffold. Hyperbranched polymer (HP) and miRNAs formed polyplexes in water. The PLGA microspheres encapsulated HP/miRNA polyplexes. The PLGA microsphere-incorporated PLLA NF scaffolds were implanted into mice to promote bone regeneration. The HP/miRNA polyplexes could be introduced into cells through endocytosis. After enzymatic polymer degradation, miR-26a was then released in the cytosol where it could perform its regulation of gene expression. Reprinted from a previous study Zhang X. et al. (2016) with permission.

For *in vivo* local bone induction, Manaka et al. (2011) used a hydrogel consisting of a poly-d, l-lactic acid-p-dioxanone-polyethylene glycol block copolymer (PLA-DX-PEG) as an siRNA carrier to increase BMP-2 expression levels and promote local new bone formation. The investigation indicated that the ectopic bone formation induced by hydrogel encapsulated with an siRNA targeting noggin (antagonist to BMPs) and BMP-2 was significantly increased when compared with those induced by hydrogel with BMP-2 alone. Moreover, the translocation efficacy of double-stranded RNA (dsRNA) from this type of hydrogel was higher than that of local injection, which indicated a promising and effective hydrogel delivery system for siRNA therapy. For miRNA delivery, Zhang X. et al. (2016) used a hyperbranched polymer (HP) vector consisting of PEG chains and a low molecular weight cationic PEI for miR-26a delivery; the miRNA was mainly encapsulated in biodegradable polymer microspheres, which self-assembled into the hydrophilic PEG layer (Figure 4). This two-stage miRNA delivery system significantly improved the miRNA release duration and transfection efficiency. In addition, the microspheres were immobilized into a nanofibrous 3D scaffold, which facilitated the localization of the scaffold and activation of endogenous cells to promote regeneration of critical-sized calvarial bone. After delivering miR-26a into the bone defect site, miR-26a was able to target *Gsk-3β* and activate the Gsk-3β/β-catenin signaling pathway in osteoblasts, which promoted osteogenic differentiation and bone regeneration. This study indicated that hydrogels could be a promising platform for controlling miRNA delivery.

Nguyen et al. (2014) synthesized an *in situ* 8-arm PEG hydrogel loaded with siRNA/PEI nanocomplexes. The siRNA release profile indicated that sustained delivery was obtained, and siRNA remained bioactive over a prolonged period. *In vitro* experiments showed that sustained delivery of siNoggin and siNoggin/miRNA-20a augmented hMSC osteogenic differentiation in hydrogel 3D cultivation (Nguyen et al., 2014). Similar results were obtained in a rat calvaria bone defect model (Nguyen et al., 2018). Bone is a highly vascularized tissue; hence, the angiogenic procedure is as crucial as osteogenesis during bone repair. Li et al. (2013) created a miRNA-26a enhancer sustained delivery system by encapsulating MSCs and an agomir into a commercialized hydrogel. *In vivo* osteogenesis experiments demonstrated that sustained release enhanced miRNA-26a expression at both the defect and the surrounding tissue, which promoted vascularization and bone formation. Huynh et al. (2016) introduced a photodegradable linkage to a PEG hydrogel matrix. Thus, intriguingly, the release of siRNA could be triggered and accelerated by ultraviolet light, which could provide “on-demand” RNA delivery. Moreover, sustained delivery of miRNA inhibitors capable of downregulating endogenous miRNA expression would also be beneficial to bone regeneration. Wu et al. (2018) constructed stromal cell-derived chitosan/tripolyphosphate/hyaluronic acid/anti-miRNA-138 nanoparticles and loaded stromal cell-derived factor-1α (SDF-1α) in a chitosan/β-sodium glycerol phosphate hydrogel for bone regeneration. The spatiotemporal sequence release pattern of the bioactive factors enhanced osteogenesis both *in vitro* and *in vivo*.

## Conclusion and Future Perspective

With the approval of patisiran, liver-targeted RNAi systemic therapy has become a clinical reality. The therapeutic effect of delivering RNAi to non-liver and non-kidney tissues has become viable in the experimental setting. For bone tissue regeneration, a range of investigations have identified the potential of hydrogels to deliver RNAi molecules to specific bone defect sites and achieve sustained gene silencing. Emerging modification methods have been carried out with hydrogel delivery systems for accelerating bone tissue regeneration, and they have shown efficacy in preclinical animal models. However, translation to the clinic is still ongoing.

The unique properties of hydrogels, such as their high water content, help to improve cell adhesion and tissue response. Their mechanical, chemical, and physical properties can also be modulated for specific clinical applications. Moreover, for drug delivery, hydrogels are an ideal platform for assisting in retention and promoting sustained drug release.

While hydrogels are a promising platform for RNAi delivery, the improvement of RNAi molecular release and uptake from hydrogels is still needed. The structure and size of nanoparticles may need more exploration to facilitate hydrogel-based delivery methods. Micro- or nanopatterning can also be used to study and understand the cellular responses to RNAi (Lee and Koh, 2014; Jeon et al., 2018; Li et al., 2019). Furthermore, a drug release signal could also be fabricated into hydrogel to improve responsiveness, such as pH (Hossieni-Aghdam et al., 2018; Shi et al., 2018), temperature (Amoli-Diva et al., 2017; Turabee et al., 2018; Wang P. et al., 2018), stress (Chaudhuri et al., 2016; Wei et al., 2018), enzymatic activity (Lian et al., 2016; Skaalure et al., 2016), and light (Song et al., 2015; Rastogi et al., 2018). An appropriate administration time might enhance the effectiveness of RNAi therapy and help to promote the clinical application of hydrogel RNAi therapy for bone regeneration.

## Author Contributions

TY and YZ collected the literature and wrote the manuscript. BH and XW conceived and revised the content of manuscript. HW collected the literature and revised the manuscript. All authors approved the final version of the manuscript for publication.

## Conflict of Interest

The authors declare that the research was conducted in the absence of any commercial or financial relationships that could be construed as a potential conflict of interest.
